# Global Positioning System Data-Loggers: A Tool to Quantify Fine-Scale Movement of Domestic Animals to Evaluate Potential for Zoonotic Transmission to an Endangered Wildlife Population

**DOI:** 10.1371/journal.pone.0110984

**Published:** 2014-11-03

**Authors:** Michele B. Parsons, Thomas R. Gillespie, Elizabeth V. Lonsdorf, Dominic Travis, Iddi Lipende, Baraka Gilagiza, Shadrack Kamenya, Lilian Pintea, Gonzalo M. Vazquez-Prokopec

**Affiliations:** 1 Program in Population Biology, Ecology, and Evolution and Departments of Environmental Sciences and Environmental Health, Emory University, Atlanta, Georgia, United States of America; 2 Division of Foodborne, Waterborne and Environmental Diseases, Centers for Disease Control and Prevention, Atlanta, Georgia, United States of America; 3 Department of Psychology, Franklin and Marshall College, Lancaster, Pennsylvania, United States of America; 4 College of Veterinary Medicine, University of Minnesota, Minneapolis, Minnesota, United States of America; 5 The Jane Goodall Institute, Kigoma, Tanzania; Linneaus University, Sweden

## Abstract

Domesticated animals are an important source of pathogens to endangered wildlife populations, especially when anthropogenic activities increase their overlap with humans and wildlife. Recent work in Tanzania reports the introduction of *Cryptosporidium* into wild chimpanzee populations and the increased risk of ape mortality associated with SIVcpz-*Cryptosporidium* co-infection. Here we describe the application of novel GPS technology to track the mobility of domesticated animals (27 goats, 2 sheep and 8 dogs) with the goal of identifying potential routes for *Cryptosporidium* introduction into Gombe National Park. Only goats (5/27) and sheep (2/2) were positive for *Cryptosporidium*. Analysis of GPS tracks indicated that a crop field frequented by both chimpanzees and domesticated animals was a potential hotspot for *Cryptosporidium* transmission. This study demonstrates the applicability of GPS data-loggers in studies of fine-scale mobility of animals and suggests that domesticated animal–wildlife overlap should be considered beyond protected boundaries for long-term conservation strategies.

## Introduction

Patterns of fine-scale animal mobility within heterogeneous landscapes have significant impact on foraging and reproductive success, inter and intra-specific competition, creation and persistence of meta-populations, gene flow and structure, and propagation of infectious diseases [1]–[6]. Mobility depends on a complex repertoire of biological, behavioral and environmental processes including animal fitness, habitat selection, dispersion, foraging, social interactions, predator aversion, and reproduction, which may occur across multiple spatial and temporal scales [7]–[10]. Within species, fine-scale mobility patterns vary with resource availability or habitat loss [11], [12].

At fine spatial scales (i.e., landscape level), animal mobility has historically been quantified by direct observation, mark-recapture studies or radio-telemetry [13]–[15]. Direct observations and mark-recapture studies are labor intensive, affected by observer bias, and not suitable for some animal systems. Radio-telemetry, which relies on animals wearing radio transmitters overcomes some of these limitations; however, these systems still require close range observations that can influence animal behavior, limit the number of animals studied, and frequency of data captured [16], [17].

Global positioning systems (GPS) determine the location of an individual on or above the Earth's surface 24 hours a day with a high level of accuracy [18]. The large size of GPS collars has limited GPS telemetry studies to large animals with few individuals [19]–[21]. However, recent reductions in the size and cost of GPS telemetry devices now make them attractive for ecological, biological and epidemiological studies of the interplay between a larger number of smaller wild and domesticated animals, their local environment, and spread of disease.

Domesticated animals play an important role in the persistence, amplification and transmission of zoonotic pathogens and serve as sources of pathogen spillover to endangered wildlife populations, especially when anthropogenic activities lead to habitat overlap between domesticated animals and wildlife [22], [23]. Epizootic diseases such as Chagas and Nipah virus infections cause morbidity and mortality in humans and wildlife; intervention strategies are hampered by the persistence of animal reservoirs, with recursive mobility and pathogen amplification capability [24], [25]. Despite the critical role that domesticated animals play in disease dynamics, a lack of suitable tools to continuously track animal mobility has hindered the study of mechanisms driving zoonotic spillover and disease emergence.

Empirical evidence indicates that the endangered chimpanzee (*Pan troglodytes schweinfurthii*) population of Gombe National Park, Tanzania, is infected with enteric parasites associated with humans and domesticated animals [26]. They have experienced SIV*cpz*-associated morbidity [27], which may be complicated by *Cryptosporidium* co-infection, as seen in human HIV-*Cryptosporidium* co-infections [28]. *Cryptosporidium* is a zoonotic gastrointestinal parasite of a wide range of vertebrates, highlighting its transmission potential between host species [29], from ingestion of contaminated food or water [30]. *Cryptosporidium* is common in ruminants and completes its life cycle in the animal's intestines, shedding high numbers of infectious oocysts into the terrestrial environment [31]. Despite these health threats, few studies have examined the ecology of this pathogen in rural tropical forest systems characterized by high rates of overlap among humans, domesticated animals, and wildlife [32].

The present study aimed to 1) assess the utility of GPS telemetry to capture the mobility patterns of domesticated animals (dogs, goats and sheep) using portable GPS data-loggers and 2) establish putative *Cryptosporidium* transmission routes by determining the extent of infected domesticated animal overlap with chimpanzees.

## Methods

### Ethics Statement

This project was reviewed and approved by the Emory University Institutional Review Board (approval #: IRB00018856) under the Expedited review process per 45 CFR 46.110(3), Title 45 CFR Subpart D section 46.404, one parent consent, and 21 CFR 56.110 and the National Medical Research Institute, Dar Es Salaam Tanzania. All animal use followed the guidelines of the Weatherall Report on the use of non-human primates in research and was approved by the TanzaniaWildlife Research Institute, the Emory University Animal Care and Use Committee (protocol ID 087-2009), and Tanzania Commission for Science and Technology (permit number 2009-279-NA-2009-184). Approval was also obtained from Tanzania National Parks (Permit number TNP/HQ/C10/13) to collect samples from wild chimpanzees. The researchers did not have any interactions with the chimpanzees at the park. All animals were sampled from households in Mwamgongo village. The owners of the animals provide verbal consent for the use of their animals for this study, and the verbal consent was documented. We have included the GPS coordinates for Mwamgongo village at the first mention of the village in the Methods section.

### Study Site

**Figure 1:**
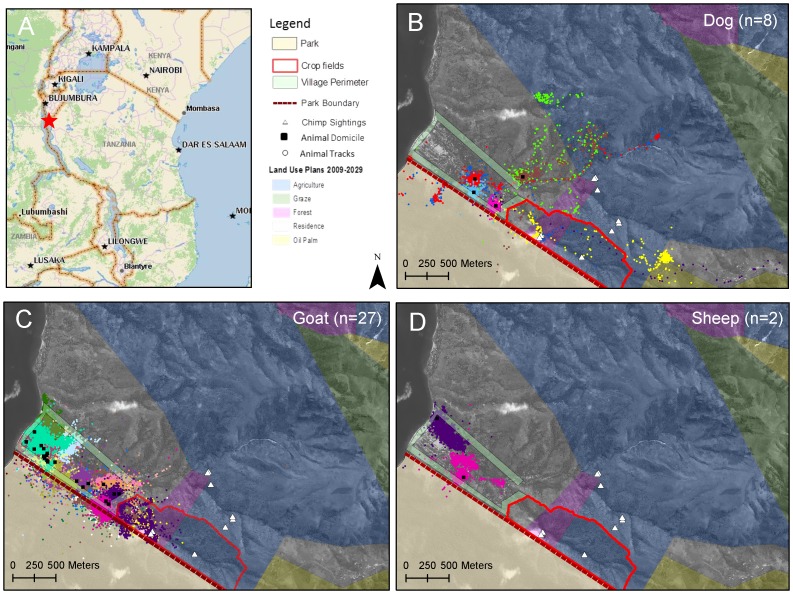
Gombe National Park and Mwamgongo Village, Tanzania. A. Location of study site within Tanzania. B-D Land use plans, chimpanzee sightings, park border, crop-raiding zone, village perimeter and animal mobility tracks of dogs, goats and sheep. Dot colors represent mobility pattern of each individual animal.

Gombe National Park, (35 km^2^) is located 16 km north of Kigoma on the shore of Lake Tanganyika in western Tanzania (4°40′S, 29°38′E) (Fig 1A), 1,500 m above sea-level [33]. Human presence in the park is restricted to researchers, tourists, park management, local field assistants, and associated families. The northern park boundary and adjacent village, Mwamgongo (4°40′S, 29°34′60′ E) are geographically isolated, accessible from Kigoma only by a 3–4 hour motorized boat trip or to the nearest villages by foot (distance 10 km; 3 hour travel time). Mwamgongo's approximately 7,000 residents are fishermen and farmers of palm oil, maize and cassava [34]. Domesticated animals include goats, sheep, Muscovy ducks, chickens and household dogs that are untethered with access to areas outside the village. There are no pigs due to religious preference. The area experiences bi-modal seasonality, with a wet season from October to mid-May with annual rainfall of 1,600 mm [35].

The park border is porous; people and animals follow the natural boundaries freely moving in and out of the park. Chimpanzee sightings have been reported east of the village in an area used predominantly for agriculture, where villagers report spreading goat feces on crops as a deterrent to crop-raiding goats and chimpanzees (I. Lipende, personal communication). We focused our study at the park boundary and in the agricultural fields east of the village as these locations are the areas with the highest potential for human-domesticated animal-chimpanzee interaction (Fig 1B-D).

### Study Enrollment and GPS Deployment

The study consisted of a demographic survey and two GPS deployment and diagnostic specimen collection periods during the dry (1 July–15 August 2010) and wet (1 November–15 December) seasons to capture seasonal variation in animal movement patterns. A baseline demographic survey was performed in June 2010 to identify households within Mwamgongo village with at least one domesticated animal species: dog (*Canis lupus*), goat (*Capra hircus*) or sheep (*Ovis aries*), and to estimate the number of domesticated animals within the village. Each homeowner was informed of the study purpose and the data the GPS units would capture and asked for consent. Homeowners provided documented verbal consent for the use of their animals in this study. Movement data was captured using GPS data-loggers (Igot-U GT120, Mobile Action Technology Inc., Taipei, Taiwan) strapped to adjustable animal collars and placed visibly around the neck. We programmed units to capture data at 2 min intervals for eight consecutive days. A GPS unit exchange was scheduled after four days for functionality check, data download, and battery recharge. Efforts were made to survey the same animals in both seasons. If the same individual was not available, then a new individual was enrolled. We collected a fecal sample when an animal was collared.

Within Gombe National Park, we non-invasively collected fecal samples from known individuals of two communities of habituated chimpanzees. There was no direct interaction with the chimpanzees in the National Park. Kasekela, the larger community, is situated at the center of the park and has been studied continuously since 1960. Mitumba, the smaller Northern community, was habituated in the mid-1990s and is in proximity to Mwamgongo. The sample set comprised of 58 members of the Kasekela community and 26 members from Mitumba. Fecal samples were tested for *Cryptosporidium* spp. by molecular methods. Briefly, total nucleic acid was extracted from fecal samples [36] using the FastDNA spin kit (MP Biomedical, Solon, OH). DNA extracts were tested by polymerase chain reaction targeting 18S gene sequences specific for *Cryptosporidium* [37]. *Cryptosporidium* prevalence was evaluated using univariate Fisher's exact tests to determine significance between groups in SPSS version 20.0 (International Business Machines, Armonk, NY USA).

### Data management and analysis

GPS data were downloaded by connecting each GPS unit via USB to a personal computer using @trip software (Mobile Action Technologies). Each individual was assigned a unique ID with data saved as.csv and.gpx files. Surveyed households were mapped by capturing a waypoint at the front door of each domicile. All experimental data was projected (UTM Zone 35S, WGS 1984 datum) and imported into ArcGIS 9.3 (ESRI, Redlands, CA). GPS mapping and local observations were utilized for park boundary demarcation and high-resolution satellite imagery (QuickBird, Digital Globe) used to determine the agricultural zone (663.5 km^2^). Raw GPS point data was used to evaluate GPS battery performance with descriptive statistics and independent two-tailed Student t-tests to evaluate significance. The positional point and line accuracy of the GPS units used in this study were previously found to be 4.4 m and 10.3 m respectively [38].

We limited analysis of mobility to data points captured during daylight hours (7:00–17:00 EAT; http://www.giasma.com/en) when animals were expected to be active. Average daily median and mean maximum distances traveled were calculated for each animal. A spatial join between points within the park and the village-park border was configured to calculate the median distance of park introgression. Data points were then aggregated to calculate the proportion of points within the park, the agricultural zone and the village perimeter (other areas outside the village but not within the park or agricultural zone). Statistical analyses were performed using Mann-Whitney U and Chi-square non-parametric tests in SPSS and SOFA Statistics version 1.3.2 (Paton-Simpson & Associates Ltd, Auckland, New Zealand) to assess differences between groups. Household mobility data was excluded from the analysis, as there have been no reports of chimpanzees entering the village.

## Results

Twenty-five households (96.1% of all contacted households) participated in the study. Sample sizes were determined as the number of animals collared over the estimated population size for each species. We collared 8 dogs (100% of village population), 2 sheep (20% of population), and 27 goats (18% of population). Longitudinal data collection was possible for all sheep, 75% of goats and 37.5% of dogs. We were unable to resample 3 individuals due to death (1 goat and 2 dogs), inability to recapture the animal (1 dog) or unavailability of household occupants (1 goat). A single household declined enrollment due to concern that their animal (goat) may get stuck in grasses. Only one GPS collar (4%) was never retrieved due to the animal (dog) being reported stolen.

In total, 123 unique animal readings were captured by GPS. Eight (6.5%) generated <24 hrs of data (range 0:00–22:00 hrs) due to programming error and were excluded from evaluation. The GPS units maintained an average battery life of 92:00 hrs (range: 27:20–123:30 hrs; (SD) = 16:40). Battery life was not affected by season with an average use of 95:50 hrs (range: 27:20–134:50) in dry season versus 89:60 hrs (range 37:40–123:30) during the wet season (*p*-value  = 0.165). If the average unit held a battery life of 92 hours, then this could generate 2,760 signals per collar rotation given that the units were programmed to capture a point every two minutes. The average number of GPS points captured was 1,634 (60% coverage). Fewer signal points were generated during the wet season (1,501 points) versus dry (1,891), (independent Student's t- test, *p*-value 0.002).

**Figure 2:**
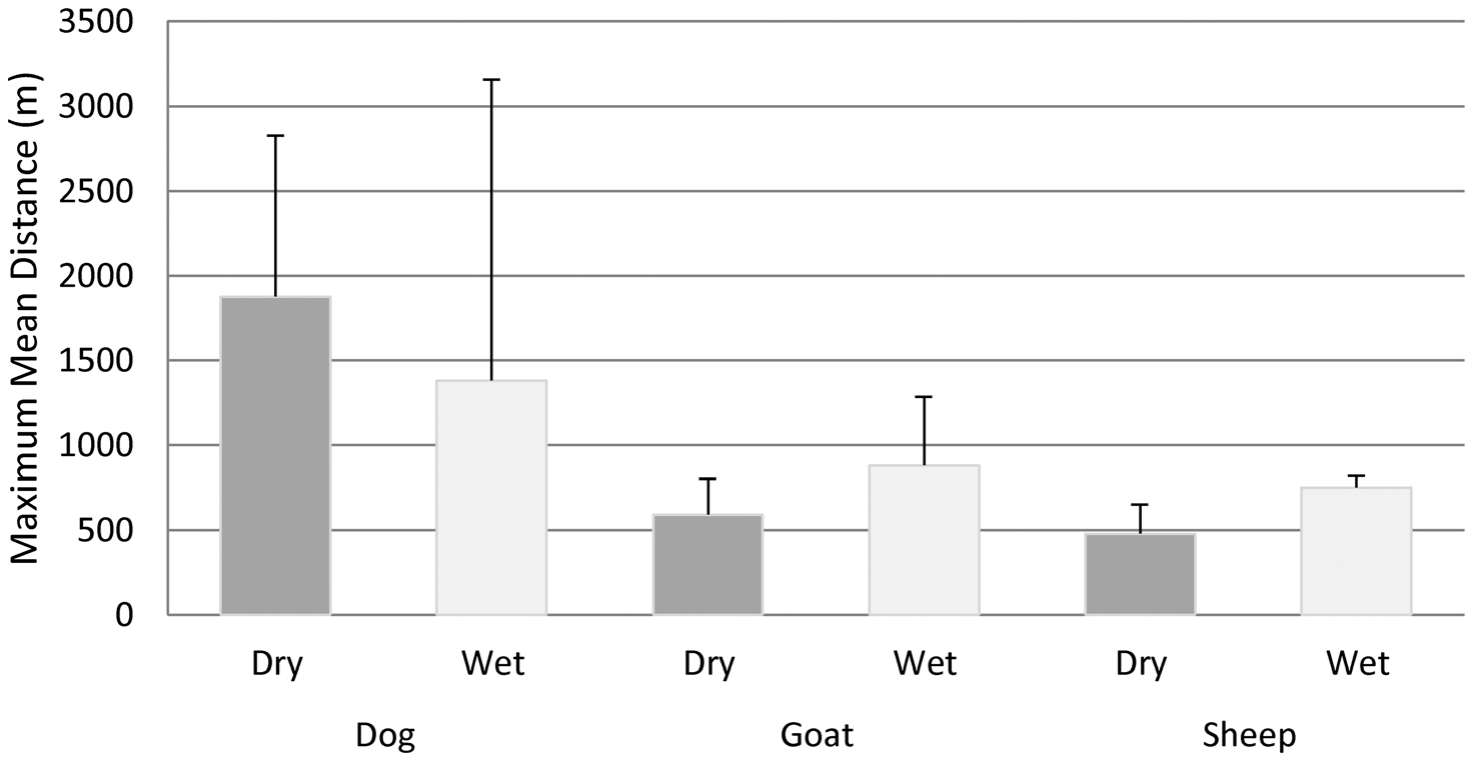
Mean maximum distance traveled from home (m) in dry and wet seasons by domesticated animal species in Mwamgongo village, Tanzania.

Five goats (18.5%) and two sheep (100%) were positive for *Cryptosporidium*. All dogs were negative for the parasite. Infection status was not determined to affect the daily median distance of sheep (Mann-Whitney U = 31, *p*-value = 0.91) or goats (Mann-Whitney U = 561, *p*-value = 0.95) from their domicile (Table 1). Goats moved significantly further from home during the wet versus dry season (U = 8,935, *p*-value <0.001) (Table 1). Small sample sizes prevented us to test whether such seasonal variation was observed in sheep or dogs. There was variation in the distance that dogs moved on a daily basis; one individual dog traveled approximately 4,500 m from its residence in the dry season compared to the canine daily median distance of 24.5 m (IQR: 15–4,565 m). Dogs also had the greatest daily mean maximum distance traveled at 1,876 m in the dry season compared to 589 m for goats and 471 m for sheep (Figure 2).

**Table 1:** Comparison of mobility patterns of domesticated animal species in Mwamgongo village adjacent to Gombe National Park, Tanzania.

Daily distance traveled from domicile (m)			
**Seasonality**	**Dry**	**Wet**	***p-*** **value**
Dog (n = 8)	24.5 (15–4564)	34 (13–2061)	*p* = 0.382
Goat (n = 27)	63 (10–432)	127 (13–909)	*p* = 0.001*
Sheep (n = 2)	204 (64–268)	185 (85–221)	*p* = 0.091
**Infectious Status**	***Cryptosporidium*** ** Negative**	***Cryptosporidium*** ** Positive**	***p*** **-value**
Goat (n = 5)	67 (17–282)	85 (10–257)	*p* = 0.095
Sheep (n = 2)	204 (64–268)	185 (85–221)	*p* = 0.091

Results are expressed as median with interquartile range in parentheses. *Statistically significant.

**Figure 3:**
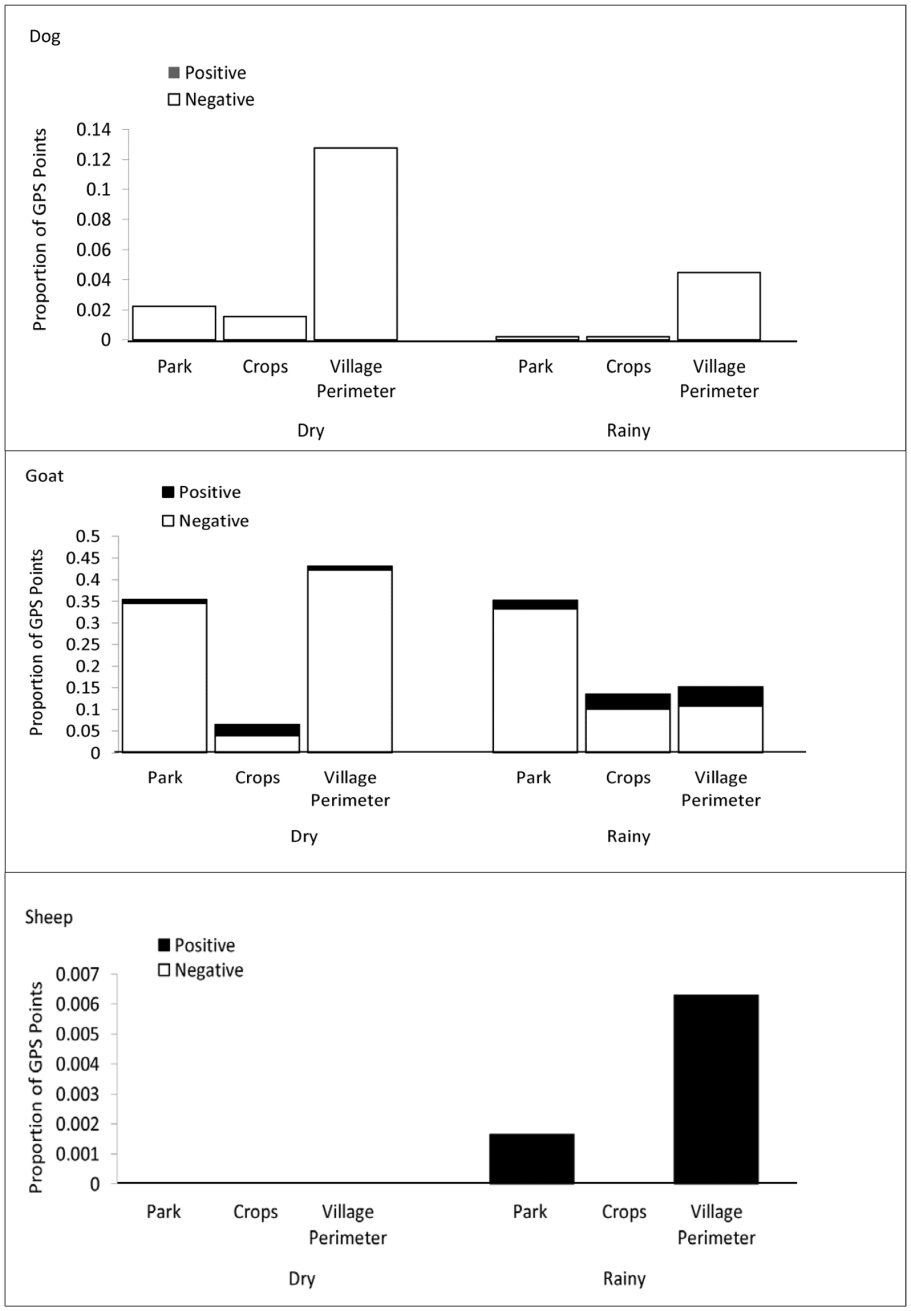
The proportion of GPS points from infected and uninfected domesticated animals in the defined study area in Mwamgongo village and Gombe National Park, Tanzania, during the dry and wet seasons. Infected animals (Goat: n = 5, Sheep: n = 2, Dogs: n = 0).

All species moved into the national park with the median distance of introgression less than 50 m (dogs: median distance 37 m, IQR 20–80; goats median distance 20 m, IQR 11–39; sheep median distance 50 m, IQR 23–196). Of the three species, goats spent the most time in the park in the dry (35% of GPS points collected) and wet (35%) seasons (Figure 3; *p*-value <0.0001 for both seasons) compared to dogs (≤2% of GPS points) and sheep (≤1%). Outside the park, the crop fields were commonly visited by dogs and goats; in the dry season, 7% of GPS points were from seven goats that visited or moved through crop fields compared to 10 goats (14%) in the wet season. Four dogs (two per season) moved into these fields during the dry (2%) and wet (1%) seasons.

Goats infected with *Cryptosporidium* did not have different ranging patterns from those that were not infected (X^2^ = 0.157, df = 1 *p-*value 0.691). The aggregated GPS data showed that of the 5 goats with a specimen yielding *Cryptosporidium*, infection status did not alter ranging behavior; three goats were found to spend time in the park but not the crops fields while either infected or uninfected. A fourth goat, though *Cryptosporidium* positive in the dry season, maintained a ranging pattern in both seasons that included the park and crop fields. One infected goat was not found in either location. Infected goats made up 40% (dry season) and 25% (wet) of GPS points of all goats in the crop fields and contributed 2.7% (dry) and 5.6% (wet) of all goats points in the park (Figure 3). Small sample size made it difficult to discern significance of sheep ranging patterns based upon infection status. Both sheep collared were positive for *Cryptosporidium* in the wet season only. One sheep moved into the park during the wet season but not the dry season. The other infected sheep stayed within the village perimeter. Neither sheep visited the crop fields. The prevalence of *Cryptosporidium* in the Mitumba and Kasekela chimpanzee communities was 15.4% and 20.6% but the difference between groups was not significant (Fisher's exact test (two-tailed) *p*-value 0.7660). Rates of chimpanzee crop raiding are unknown since monitoring does not occur outside park boundaries but villager sightings of Mitumba chimpanzees raiding the crop fields occur year round with greater frequency reported in the dry season.

## Discussion

The human-wildlife interface is permeable and regularly altered by relatively unknown ecological and epidemiological dynamics. Our study demonstrates that portable GPS technology allows rapid and concurrent characterization of fine-scale movement from multiple individuals in a population. This technology, in combination with clinical data, can also be used to identify the spatial arrangement of those individuals infected with an etiologic agent at a specific point in time, and determine whether their infection status may alter fine scale movement processes and/or be used to predict probabilities of disease spread over time. The GPS unit cost, battery life, durability, size and weight made it suitable for field deployment. The affordability of the unit ($50 USD each) allowed for tracking 42 animals (up to 12 animals per exchange) simultaneously for two consecutive 96-hour periods. Cost is a limiting factor when considering study design and number of individuals to sample, and GPS data-loggers like the ones used in this study can provide a reliable way for tracking fine-scale local movements of domesticated animals.

The size (L/W/H in mm; 44.5×28.5×13) and weight (20 g) of the units minimized the likelihood of influencing animal movement and behavior and allows for the tracking of smaller animals. The durable, water resistant properties of the unit made it suitable for tracking during excessive rainfall. Strapping the unit to an adjustable, clip-on collar minimized investigator contact with study animals, thereby reducing stress. The low refusal rate by households invited to participate in the study indicated limited apprehension to the units and associated data collection. We collected 117 unique animal readings representing over 10,500 hours of animal mobility data. The only performance difference detected was the collection of fewer GPS points during the wet season, potentially due to factors affecting satellite signal loss such as greater canopy cover, excessive rainfall, or atmospheric conditions [39].

The GPS data provide unique insights on the patterns of domesticated animal mobility and potential interaction with chimpanzees within the park and crop-raiding areas. Our study suggests that goats, due to their high overlap with chimpanzee habitats, are the most likely spillover host and that crop-raiding areas are the potential *Cryptosporidium* spillover hotspot into chimpanzee populations. The frequency of habitat overlap during the dry season is compelling as chimpanzees are more likely to raid crops as they seek out alternative food sources. Crop raiding is a frequently reported behavior among primates [40]. A Nigerian study reported that crop-raiding baboons were more likely to harbor the anthropogenic parasite *Balantidium coli* as compared to their less frequent raiding counterparts [41]. Interestingly, Gillespie et al. [26] found that *B. coli* only occurred in Mitumba chimpanzees and that overall chimpanzee parasite diversity was higher for Mitumba chimpanzees compared to Kasekela chimpanzees. During the study period, it was also reported that villagers may spread animal feces on crops as a natural deterrent (I. Lipende, personal communication), which has the potential to indirectly perpetuate the spread of infection in this identified environmental hotspot.

*Cryptosporidium* was detected in both Mitumba and Kasekela but there was no significant difference in the prevalence of *Cryptosporidium* between groups. Although not statistically significant, it was surprising to find a moderate prevalence of *Cryptosporidium* in Kasekela community as compared to Mitumba. The Kasekela chimpanzees have less interaction with humans and no interaction with livestock as compared to the Mitumba community whose natural border with Mwamgongo village places them in contact with human and animal activities and at greater risk for zoonotic infections. These results suggest that different transmission cycles are operating in these groups. Additional molecular characterization of the positive samples is underway to determine the species of *Cryptosporidium* in this system to assess potential transmission pathways and sources for exposure.

*Cryptosporidium* is of global concern [28] and capable of surviving in the environment for long periods. The parasite has been detected in other habituated primates. A bovine genotype was isolated from gorillas and people living in and around Bwindi Impenetrable National Park, Uganda [42], [43]. Salyer et al. [44] examined the molecular epidemiology of *Cryptosporidium* in wild primates, people and livestock in and around Kibale National Park in Uganda and found red colobus (*Procolobus badius*) and black-and-white colobus (*Colobus guereza*) infected with *Cryptosporidium parvum/C. hominis* that resembled that of people and livestock at the forest edge, while red colobus in the forest interior were infected with a divergent subclade, suggesting the possibility of separate zoonotic and sylvatic cycles. These findings suggest that zoonotic transmission of *Cryptosporidium* can be frequent and occur with ease in tropical settings where people, livestock, and wild primates overlap.

Sheep were positive for *Cryptosporidium*, but their movement was predominantly restricted to the village, limiting their probability of contact with chimpanzees. It is of note that only two of the village sheep were tracked and their mobility patterns may not represent movement of all sheep in the village. Although the dogs tracked in this study were all negative for *Cryptosporidium,* they demonstrated substantial overlap with chimpanzees and may present other zoonotic risks to chimpanzees, such as rabies, foot and mouth disease and parasitic worms.

The findings from this research have local and broad implications. Although there was evidence of animal mobility into the park, the few animal points beyond the forest edge indicate limited park intrusion. Rather, the area where chimpanzees are known to raid crops was a potential hot spot for pathogen spillover due to high visitation rates by domestic animals and the dispersion of potentially infected animal feces. Application of animal feces to crops may perpetuate the transmission of foodborne pathogens [45]. Pathogen introductions into the environment can occur in both the dry and wet seasons but actual spillover to chimpanzees was more likely to occur in the dry season.

The focus of conservation strategies traditionally has been on wildlife corridors to minimize habitat fragmentation and risk for population isolation [46]. These data suggest a need to monitor borders and edges, in wildlife management and park design. In this case, the application of integrated GPS/GIS technology was capable of identifying a novel area along the Mwamgongo/Gombe border most at risk from human and animal use that may impact the sustainability of vulnerable wildlife populations.
